# Randomized experimental testing of new survey approaches to improve abortion reporting in the United States

**DOI:** 10.1363/psrh.12217

**Published:** 2022-12-13

**Authors:** Laura D. Lindberg, Isaac Maddow‐Zimet, Jennifer Mueller, Alicia VandeVusse

**Affiliations:** ^1^ Department of Urban‐Global Health Rutgers School of Public Health (formerly at the Guttmacher Institute) Piscataway New Jersey USA; ^2^ Research Division Guttmacher Institute New York New York USA

## Abstract

**Context:**

Abortions are substantially underreported in surveys due to social stigma, compromising the study of abortion, pregnancy, fertility, and related demographic and health outcomes.

**Methods:**

In this study, we evaluated six methodological approaches identified through formative mixed‐methods research to improve the measurement of abortion in surveys. These approaches included altering the placement of abortion items in the survey, the order of pregnancy outcome questions, the level of detail, the introduction to the abortion question, and the context of the abortion question, and using graduated sensitivity. We embedded a preregistered randomized experiment in a newly designed online survey about sexual and reproductive health behaviors (*N* = 6536). We randomized respondents to experimental arms in a fully crossed factorial design; we estimated an average treatment effect using standardized estimators from logistic regression models, adjusted for demographic covariates associated with reporting.

**Results:**

None of the experimental arms significantly improved abortion reporting compared to the control condition.

**Conclusion:**

More work is needed to improve reporting of abortion in future surveys, particularly as abortion access becomes increasingly restricted in the United States. Despite this study's null results, it provides a promising path for future efforts to improve abortion measurement. It is proof of concept for testing new approaches in a less expensive, faster, and more flexible format than embedding changes in existing national fertility surveys.

## INTRODUCTION

Pervasive abortion underreporting in individual‐level surveys compromises the study of abortion, pregnancy, and fertility by undermining the data on which many public health and policy decisions depend.^1^, ^2^ Despite its frequency, abortion remains a highly sensitive and thus difficult‐to‐measure behavior across a range of settings.^3^, ^4^, ^5^ Past studies have documented widespread abortion underreporting by respondents in multiple surveys in the United States for decades,^6^, ^7^, ^8^ and the National Survey of Family Growth (NSFG), a premier national survey of pregnancy data, explicitly discourages using the abortion data it collects because of incomplete reporting.^9^ For instance, Lindberg and colleagues recently estimated that women reported only 30%–40% of their abortions in the National Survey of Family Growth (NSFG), the National Longitudinal Survey of Youth (NLSY), and the National Longitudinal Study of Adolescent to Adult Health (Add Health); the completeness of reporting varied across demographic groups, but substantial underreporting was universal.^10^ Desai and colleagues estimated that almost 11% of pregnancies are missing from the NSFG due to abortion underreporting; the share increased to 18% among unmarried women and Black women.^1^ Differential abortion underreporting and missing pregnancies bias many analyses of sexual and reproductive health, impacting not only studies that focus on abortion but also those examining pregnancy more generally.^1^, ^2^, ^10^ The lack of data on individuals' abortion experiences is a significant roadblock to efforts to improve public health and advance reproductive autonomy. Thus, improving the measurement of abortion in surveys is imperative.

Abortion underreporting reflects pervasive abortion stigma in the United States and elsewhere.^11^, ^12^, ^13^ Abortion stigma has been defined as “a negative attribute ascribed to [people] who seek to terminate a pregnancy,” and experiences of stigma are often tied to gendered and racialized societal expectations of behavior.^11^ People who have sought abortions may choose not to disclose this information to others to avoid judgment and condemnation.^14^, ^15^ In the Turnaway Study, perceived stigma was reported by more than half of a sample of more than 900 people who sought an abortion.^16^ In particular, participants said that they anticipated that “people in their community and people close to them would look down on them if they knew they had sought an abortion,” mirroring findings in prior research.^17^, ^18^ Abortion concealment as a stigma‐avoidance strategy may lead people to underreport abortions in surveys.

Abortion stigma may make reporting this behavior particularly sensitive in a survey context. Tourangeau and Yan argue that misreporting of sensitive behaviors is primarily deliberate and a form of “motivated misreporting”^19^; a survey item may be “sensitive” because it raises concern about social desirability, is intrusive, or disclosure risks negative consequences for the respondent.^20^ Questions can be sensitive for multiple reasons and each type of sensitivity may influence survey responses for self‐reporting abortion. In addition, research on the survey response process suggests that some abortion misreporting could also be due to comprehension issues if respondents are unclear about what experiences to report as abortion^21^; qualitative research indicates this can be an issue.^22^

Despite a history of abortion measures in many U.S. surveys (recent national surveys that ask about abortion include NSFG, Add Health, NLSY, the Panel Study of Income Dynamics [PSID], the Fragile Families and Child Wellbeing Study, and the National Health and Nutrition Examination Survey [NHANES] as well as local surveys such as the Toledo National Relationship Study and the Relationship Dynamics and Social Life [RSDL]), there is limited documentation of methodological innovations to improve reporting. As early as its first cycle in 1973, the NSFG tried to collect data on abortion and found the reports unreliable.^23^ In the 1990s, the NSFG conducted experimental testing of different abortion measurement approaches to improve the methodology. This testing included different survey modes, question‐wording, question order, changing the interview location, increasing incentives and even having nurses in uniform conduct the interviews—all with generally null results.^24^, ^25^ A critically important innovation was the addition of an audio computer‐assisted self‐administered interview (ACASI) to measure abortion and a limited set of other sensitive behaviors that also face reporting problems in the 1995 NSFG; this reflected the idea that providing more privacy by removing the interviewer would improve respondents' willingness to answer sensitive questions.^24^ This approach elicited abortion reports from some additional women, but abortion remained substantially underreported.^6^ Since 1995, there have been only modest changes to relevant abortion question items in the NSFG.^9^, ^26^, ^27^

Efforts outside the United States have often relied on indirect estimation methods or methods that obscure individual respondents' answers (such as the list experiment); however, these approaches generally can only estimate overall prevalence, as they cannot be linked to individual women's reports.^3^, ^28^ These methods have had inconsistent results and few U.S. applications exist.^29^, ^30^, ^31^ European studies have made some efforts to alter question‐wording or order to improve abortion, but with limited impact.^4^, ^5^ Given the persistent problems of substantial abortion underreporting and minimal advances in abortion measurement in the United States in more than two decades, we designed several new approaches to eliciting abortion reporting and tested each in an individual‐level national survey. These approaches included changes to both the design and placement of abortion question items, as these components have been shown to influence the reporting of sensitive behaviors.^19^, ^32^ We concentrated on altering the sensitivity of questions about abortion and thus the related social desirability bias, the sense of intrusiveness of asking about abortion, respondents' motivation to report, or respondents' comprehension or judgment of which experiences to report as abortion.

## METHODS

### Development of experimental approaches

We adopted a general process identified by Groves and colleagues to develop, refine, and evaluate new survey measures.^21^ We conducted foundational mixed‐methods research that included a series of quantitative analyses identifying the scope and correlates of abortion underreporting,^10^, ^33^, ^34^ expert advisory input, cognitive interviews with 64 women,^22^ and a pilot survey of 2000 women to evaluate possible question items.^35^ Each of these activities was informed by prior research on abortion stigma and methodological research on survey design for eliciting responses on questions addressing sensitive topics. These foundational efforts, which we have described in detail elsewhere, served as the basis for selecting the final survey approaches for experimental testing.

### Data

Data for this study came from the 2021 Guttmacher Survey of Reproductive Health Experiences (GSRHE), an online survey. NORC at the University of Chicago managed survey recruitment and fielding. NORC recruited study participants through a dual‐sampling approach to maximize sample size using AmeriSpeak®, a probability‐based panel designed to be representative of the U.S. household population and additional respondent recruitment through a non‐probability online opt‐in panel. We selected the sample from the AmeriSpeak® panel using sampling strata based on age, race/ethnicity, education, and gender. The non‐probability‐based online sample used enrollment targets for age, race/ethnicity, and education and sent e‐mail invitations, phone alerts, banners, and messaging on panel community sites to include people with diverse motivations to participate in the research. Other studies have used this combined NORC sample approach.^36^, ^37^, ^38^

We limited eligibility criteria to those assigned female at birth, age 18–49, residing in a U.S. household at the time of the survey, who have ever had penile–vaginal sex, and who could complete surveys in English. We asked respondents who matched the eligibility criteria and agreed to participate in a research study to complete an online survey. NORC applied cleaning rules to the survey data for quality control and removed 474 participants who gave responses indicative of speeding through the survey, including skipping more than 50% of survey questions, straight‐lining responses to grid questions, or answering open‐ended questions with gibberish. The final sample consisted of 6536 respondents, with 3170 probability respondents and 3366 nonprobability respondents.

In addition to asking about abortion, the survey included sections on demographics, pregnancy intentions, sexual activity, contraceptive use, use of contraceptive services, and COVID‐related experiences. The median completion time for the survey was 12 min. Participants could skip any survey questions and could choose to end the survey at any time. The survey did not collect any identifying information. We offered respondents were offered a nominal incentive for completing the survey. The Guttmacher Institute Institutional Review Board and the NORC Institutional Review Board independently approved the study procedures.

### Experimental approaches

Building from our foundational mixed‐methods research,^35^, ^39^ we identified six experimental approaches to test in a newly designed national survey. The first experimental approach tested different placements of the abortion question item within the survey. Changes to the question design included altering the order of pregnancy outcome questions, the level of detail requested, the introduction to the abortion question, the context of the abortion question, and questions using graduated sensitivity. We compared the experimental conditions for question design to a control condition mirroring the current NSFG question‐wording; there was no explicit control condition for question placement. Below we describe each approach and an overview of the motivation for its use; we present complete questions in Table A1.

#### Placement

We hypothesized that altering the placement of the abortion measures within the survey could reduce sensitivity and improve reporting.^40^ Respondents may be concerned that answering affirmatively to a question on abortion will result in intrusive follow‐up questions or extend the interview length.^4^, ^33^ For this reason, generally researchers agree that sensitive items should be asked later in a survey.^41^ We tested placing abortion measures at the very end of the survey, with an introduction that reads, “These last couple of questions are the final questions in the survey. Thank you for your participation!” This layout implicitly indicated that there would be no further question items, regardless of their response. We hypothesized that this approach could reduce any tendency to underreport abortion experiences to limit the burden of follow‐up questions. We compared reporting under this placement approach with question placement earlier in the survey, after questions on background, pregnancy intentions and contraceptive use.

#### Question‐design, control

As a control condition for the different question design approaches, we adapted the 2015–2019 NSFG ACASI pregnancy outcomes question series. In this approach, respondents first read an introduction designed to solicit more accurate reporting that explicitly acknowledges that some respondents feel reluctant to tell an interviewer about some of their pregnancies. Next, survey questions asked respondents to report their number of pregnancies that ended in a live birth, miscarriage, or abortion in three separate questions, in that order. While the NSFG asked respondents to report on pregnancies in the 5 years preceding the survey, we adapted the items to ask about lifetime pregnancies. This increased the proportion of respondents who could potentially report an abortion and, as a consequence, increased statistical power (see more details in Section 2.5). The full NSFG question wordings are available in Table A1.

#### Order

Another question design we tested was to alter the order of the pregnancy outcomes respondents are asked to report. The control condition asked about abortion after asking about pregnancies ending in miscarriage. Instead, we flipped this order and asked respondents about pregnancies ending in abortion before asking about miscarriages. In cognitive interviews,^39^ we found that some respondents expressed confusion or uncertainty about the distinction between some miscarriage and abortion experiences, a concern noted previously by other researchers^5^, ^42^; other work has speculated that respondents may be deliberately misclassifying abortions as miscarriages due to stigma.^6^, ^8^, ^22^, ^43^, ^44^, ^45^, ^46^ We hypothesized that this revised question order could reduce respondents' likelihood of intentionally or inadvertently misreporting a pregnancy as ending in miscarriage instead of abortion.

#### Level of detail

We reduced the level of detail asked of respondents about their abortion experiences compared to the control condition. We tested asking a separate yes/no question about whether a respondent ever had an abortion, followed by the more detailed question requesting the number of abortions for those who respond affirmatively. This approach contrasted with the control condition, which used a single question to ask respondents to report their number of abortions with zero included as a specific response. Asking respondents to report a specific number of sensitive events has been shown to reduce reporting quality in other measures.^32^ We hypothesized that shifting the level of detail could improve abortion reporting by reducing the perceived intrusiveness and the social desirability of the questions, as people with multiple abortions may feel greater stigma.^45^ Our cognitive interviews found that respondents prefer answering a yes/no question instead of the number of abortions due to concerns of being judged for having had multiple abortions.^22^

#### Introduction

We tested a new introduction before the pregnancy outcome; well‐designed introductions are a potential tool for improving reporting of sensitive behaviors.^19^, ^46^ The NSFG has tried different introductions over time but not in an experimental format.^47^, ^48^ In the cognitive interviews, we asked respondents whether they would want to see an introduction before a question about their abortion history. The majority felt that an introduction should be present to ease respondents into the question and explain the question motivation. However, many respondents did not like the current NSFG “reluctant” introduction; respondents often felt that the language and terminology used were negative and might discourage reporting. Instead, we explored five alternative introductions in cognitive interviews and the pilot survey. These studies found that respondents most preferred an introduction that focused on how sharing their abortion experience in a survey could help improve health services for other women.^35^ This informed the design for this study of an introduction that uses the “helping” framework. We hypothesized this introduction could improve reporting by increasing survey participants' motivation to respond accurately and help others.^19^

#### Context

Another approach was changing the context in which the survey asks respondents about abortion to reduce the effect of social desirability bias. For this approach, we asked respondents about abortion in the context of their receipt of other sexual and reproductive health care received in their lifetime. Respondents were presented with a set of health care experiences, including abortion, and asked to report yes/no for receipt of each type of care. In cognitive interviews, respondents liked this approach the most of the ones we explored.^35^ This approach was an adaptation of a question sequence already used in the NSFG, which asks about abortion in the last 12 months as part of a battery of other sexual and reproductive health care received during that period. We altered the NSFG question to consolidate the items, streamline wording, and ask about lifetime experience.

#### Graduated sensitivity

This approach attempted to desensitize abortion reporting by first asking about a range of abortion‐related experiences, including abortions of friends or family members, before asking respondents to report their own experiences. We hypothesized that this might improve respondents' willingness to report their abortions since asking related experiences can improve reporting of similar items in surveys.^49^ Additionally, some of the improvement in abortion reporting in the ACASI mode of the NSFG may be a consequence of the reduced sensitivity after the interviewer asks about abortion in prior (face‐to‐face) portions of the survey.^33^ Asking about others' abortions in the lead‐up to the question leverages findings from previous research that disclosing someone else's abortion is less sensitive than sharing one's own.^50^

### Randomization to experimental factors

We randomized respondents to receive questionnaires containing different approaches—or “treatments”—using a factorial design detailed in Table 1. Factor 1, with two levels, varied question placement in the survey instrument: abortion measures as a final question in the survey versus placement early in the survey (after questions on background, pregnancy intentions and contraceptive use). Factor 2, with six levels (including a control condition), varied the question‐design. The survey fully crossed each of the two Factor 1 levels with all six levels of Factor 2. We randomly assigned each respondent to one of these 12 conditions, with at least 500 respondents in each arm. This randomization resulted in half of the sample (~3200 in 1A and ~ 3300 in 1B) in each Factor 1 level (placement) and at least 1000 respondents in each Factor 2 level (question‐design).

**TABLE 1:** Factorial design for abortion questions in the 2021 GSRHE

Placement (Factor 1: two levels)	Question wording (Factor 2: six levels)
1A: Early	2A: Control
1B: Final	2A: Control
1A: Early	2B: Order
1B: Final	2B: Order
1A: Early	2C: Level of detail
1B: Final	2C: Level of detail
1A: Early	2D: Introduction
1B: Final	2D: Introduction
1A: Early	2E: Context
1B: Final	2E: Context
1A: Early	2F: Graduated sensitivity
1B: Final	2F: Graduated sensitivity

In each factor, the primary outcome was a dichotomous variable indicating if the respondent reported ever having had an abortion in their lifetime. (We coded the 16 respondents who skipped the relevant abortion question as having not had an abortion to avoid bias due to differential missingness. Nine respondents skipped the abortion question in the late placement, compared to seven in the early placement.) In addition to lifetime measures, in each experimental arm respondents who report an abortion received a follow‐up question asking about abortions in the last 5 years.

We estimated the target sample size in each arm based on a simulation‐based power analysis (see details in pre‐registration at https://osf.io/ypvdu). The goal was to be able to detect an increase of approximately five percentage points between a given level of Factor 2 and its control condition at 80% power while controlling the false discovery rate at 5%. The power analysis focused on Factor 2 as, with six levels, it has a smaller sample size for each level than Factor 1 with only two levels.

### Analysis

We conducted all analyses using Stata 17.0. All analyses were preregistered at https://osf.io/ypvdu before survey fielding. We first calculated the prevalence of abortion reporting for the overall sample and each factor level. These are unweighted and should not be treated as population estimates. We then tested for differences between arms in the dependent variable (respondent reports ever having had an abortion) using the standardized estimator proposed by Moore and van der Laan.^51^ This estimates the average treatment effect (ATE) using a logistic regression model adjusting for selected baseline covariates that are predictive of the outcome to improve precision.^51^, ^52^ In this case, the ATE corresponds to the marginal risk difference between arms—the difference in the predicted probability of respondents reporting an abortion for each factor level compared with the control condition for that factor level. We identified any arm resulting in higher reporting levels than the control condition as a successful survey strategy. Because of the high sensitivity of abortion reporting and prior research, we do not expect overreporting of abortion for any experimental conditions.

To improve the precision of our estimates, we adjusted for demographic characteristics known from prior research to be associated with the likelihood of having had an abortion or with the likelihood of reporting.^10^, ^33^, ^53^ We also adjusted for sampling mode, as those who opt‐in to an online sample might have a higher baseline probability of reporting sensitive behaviors. The independent variables in the model were Factor 1 groups, Factor 2 groups (with control as reference category), 5‐year age groups, race/ethnicity (non‐Hispanic white, non‐Hispanic Black, Hispanic, non‐Hispanic other race or multiple races), marital status (married, cohabiting, single), and sampling mode (probability‐based sample, opt‐in sample).

For the primary analysis, we analyzed the main effects of each treatment, assuming no interactions between Factor 1 and Factor 2. For Factor 2, we used one‐tailed tests, as we were only interested in improvements in abortion reporting over the control condition^54^; for Factor 1, we used two‐tailed tests, as neither placement (beginning or end) exactly matches the control condition for the NSFG. We also present unadjusted differences between factor levels. Given the multiple comparisons conducted, we adjusted *p*‐values using the Benjamini–Hochberg procedure, setting *q* at 0.05.^55^, ^56^ Since we were interested in treatment effects rather than population‐level parameters for this portion of the analysis, the analyses are unweighted; conclusions do not change when we applied survey weights. All code and data needed to reproduce the primary analysis are available at https://osf.io/439bu/.

In exploratory analyses, we tested for potential heterogeneity in treatment effects by introducing interactions between demographic covariates and each factor level, as well between each Factor 1 and Factor 2 level. Although specified in our pre‐registration, we label these analyses as “exploratory” because the study was not adequately powered to assess less than substantial interaction effects; in addition, we do not adjust for multiple comparisons for these tests.

We also investigated if any of the experimental approaches improved reporting of more recent abortions, given that these may be more stigmatized than less proximate experiences. We estimated the reported number of abortions in the last 5 years in each condition using the follow‐up questions about this time period. We tested for differences between experimental conditions in the likelihood of reporting any abortion in the past 5 years, and among those reporting any abortion, the likelihood of reporting one versus multiple abortions.

There are no available estimates of the true proportion of the population that has had an abortion in their lifetime. We instead benchmark the 2021 GSRHE abortion measures by comparing them to two types of NSFG reports to determine how the online survey compares to the standard NSFG interviews. First, we estimated the weighted prevalence of lifetime abortion (pooling all experimental conditions), as well as testing for differences between the probability and non‐probability samples; NORC constructed the weights to combine the probability panel and non‐probability online interviews. NORC derived weights for the AmeriSpeak panel by adjusting for the probability of selection and nonresponse, and then adjusting to population benchmarks. Their TrueNorth calibration approach uses small area estimation methods to adjust the weights for the non‐probability sample to bring the weighted distributions in line with the population distribution of characteristics correlated with the survey variables. A similar approach has been used in other studies.^37^, ^57^

We then compared the 2021 GSRHE weighted lifetime estimate to lifetime reports in the 2015–2019 NSFG face‐to‐face interview. Since the 2021 GSRHE was conducted online without an interviewer present, the NSFG ACASI reports may be a more relevant comparison; because of this we also compared the 2021 GSRHE to the 2002 NSFG ACASI, which was the most recent survey in which the ACASI mode was used to collect lifetime abortion reports. (Any differences between the 2021 GSRHE and the 2002 NSFG ACASI could also be due to declining abortion rates in the intervening years; however, lifetime abortion incidence should be somewhat less affected by period changes in rates.)

## RESULTS

### Characteristics of sample

Table 2 describes the unweighted 2021 GSRHE sample composition. The 2021 GSRHE has a substantial number of respondents in every age group, except for 18–19‐year‐olds. Slightly more than half of the respondents were non‐Hispanic white (56%) and more than two‐thirds were married or cohabitating (45% and 15%, respectively). The 2021 GSRHE is close to evenly split by recruitment mode. The distribution of socio‐demographic characteristics was similar across the 12 study arms (see Table A2).

**TABLE 2:** Percentage distribution of respondents by demographic characteristics, 2021 GSRHE (*N* = 6536)

Characteristic		%	*n*
Age	18–19	2	157
	20–24	11	737
	25–29	17	1106
	30–34	20	1314
	35–39	20	1298
	40–44	16	1075
	45–49	13	849
Race/ethnicity	Non‐Hispanic White	56	3689
	Non‐Hispanic Black	17	1082
	Hispanic	16	1034
	Non‐Hispanic other/multiple races	11	731
Marital status	Married	45	2972
	Cohabitating	15	1007
	Single	39	2557
Recruitment mode	Probability	49	3170
	Nonprobability	51	3366

*Note*: Sample selection was limited to respondents who were aged 18–49, were assigned female at birth, and had ever had penile–vaginal sex.

### Effects of question‐wording and placement

The proportion of respondents reporting abortions under each experimental condition and the associated ATE are shown in Table 3. Question placement (Factor 1) had no impact on the likelihood that respondents reported an abortion: the proportion reporting was 16% for both early and late question placement in the surveys, with an estimated ATE of 0.00 (95% confidence interval [CI], −0.02 to 0.02; adjusted *p*‐value, 0.97).

**TABLE 3:** Proportion of respondents reporting abortions under each experimental condition, and associated average treatment effect (ATE) of each experimental condition, along with *p*‐values adjusted for multiple comparisons

	%	ATE	95% CI		Adjusted *p*‐value
Question placement					
Early (ref)	16				
Final	16	0.00	−0.02	0.02	0.97
Question wording					
Control (ref)	17				
Order	18	0.01	−0.02	0.04	0.97
Level of detail	17	0.00	−0.03	0.03	0.97
Introduction	14	−0.02	−0.05	0.01	0.97
Context	14	−0.03	−0.06	0.00	0.97
Graduated sensitivity	16	0.00	−0.03	0.03	0.97

*Note*: ATEs are standardized estimators from logistic regressions adjusted for age, race, marital status, and recruitment method.

Similarly, none of the experimental question‐wording arms (Factor 2) significantly improved reporting compared to the control condition. The proportion of respondents reporting ever having had a lifetime abortion is 17% in the control condition and ranged from 14% to 18% for the other arms, with ATEs ranging from −0.03 to 0.01 (and all adjusted *p*‐values at 0.97, the theoretical upper bound of the test after applying the Benjamini–Hochberg procedure).

### Exploratory analyses

#### Comparison to the NSFG

In the weighted 2021 GSRHE sample, 16% (CI, 15%–17%) of respondents reported ever having had a lifetime abortion, compared with 13% (CI, 11%–14%) in the face‐to‐face portion of the 2015–2019 NSFG. In contrast, 20% (CI, 18%–21%) of NSFG respondents reported a lifetime abortion in the 2002 NSFG ACASI (Table 4). Some differences could be due to varying demographic compositions between surveys; even after applying weights, the 2021 GSRHE analytic sample is more highly educated than a similarly defined sample in the 2015‐2019 NSFG, although similar in regard to age, economic status, race/ethnicity, marital status, and sexual orientation. However, the demographic differentials in abortion reporting in the 2021 GSRHE parallel those in prior analyses of the NSFG and surveys of abortion patients.^10^, ^58^ 2021 GSRHE respondents were more likely to report a previous abortion if they were older than age 20–24, non‐Hispanic Black or Hispanic compared to non‐Hispanic White and unmarried compared to married. There was no significant difference in the report of an abortion in the probability and nonprobability samples of the 2021 GSRHE sample (results not shown).

**TABLE 4:** Weighted percentage of respondents who reported ever having an abortion by demographic characteristics, 2021 GSRHE, 2015–2019 National Survey of Family Growth (NSFG) and 2002 NSFG

Characteristic		2021 GSRHE (*N* = 6536)			2015–2019 NSFG (*N* = 9843)			2002 NSFG (*N* = 7643)		
		Wtd %	95% CI		Wtd %	95% CI		Wtd %	95% CI	
Total		16	15	17	13	11	14	20	18	21
Age	18–19	11	6	19	1	0	1*	7	5	11*
	20–24 (ref)	10	7	13	6	4	8	12	10	14
	25–29	12	10	15	11	9	13*	18	16	21*
	30–34	16	13	18*	10	9	12*	21	19	24*
	35–39	18	15	22*	15	13	18*	23	20	26*
	40–44	21	17	24*	16	13	19*	25	20	31*
	45–49	20	16	24*	20	16	23*			
Race/ethnicity	Non‐Hispanic White (ref)	13	12	14	11	9	12	18	15	20
	Non‐Hispanic Black	21	18	25*	23	19	27*	29	26	33*
	Hispanic	20	17	24*	11	9	13	18	16	20
	Non‐Hispanic other/multiple races	16	12	20	14	11	19	26	19	33
Marital status	Married (ref)	14	12	16	11	9	13	18	16	21
	Cohabitating	18	15	21*	16	13	19*	22	18	26
	Single	18	16	20*	14	12	16	21	19	23*

*Note*: 2021 GSRHE sample selection was limited to respondents who were aged 18–49, were assigned female at birth, and had ever had penile–vaginal sex. 2015–2019 NSFG sample includes female respondents aged 18–49 who had ever had penile–vaginal sex. 2002 NSFG sample includes female respondents aged 18–44 who had ever had penile–vaginal sex.

* Statistically significant difference at *p* < 0.05 compared to the reference category.

#### Sampling mode

There was no direct effect of the sampling mode (probability vs. nonprobability) on the likelihood of abortion reporting (see Table A3).

#### Interactions

There were no significant interactions between the question placement (Factor 1) and question‐wording (Factor 2) components of the survey instrument (results not shown). Additionally, tests for interactions between these factors and each demographic variable show no significant heterogeneous effects (results not shown).

#### Reporting abortion in the prior 5 years

Overall, 6% of respondents reported an abortion in the last 5 years in response to a follow‐up question; this did not differ between the experimental arms. Among the 1052 respondents who reported an abortion in their lifetime, 37% reported having at least one abortion in the last 5 years; among this group, 71% had one abortion and 29% had two or more. None of the experimental arms differed from the control group in the likelihood of a multiple vs. single abortion in the last 5 years (results not shown).

## DISCUSSION

This study tested experiments to improve survey respondent reporting of abortion experiences. We developed new approaches from an extended formative research process incorporating quantitative analyses of multiple national surveys, cognitive interviews, a large pilot study and input and review from experts in survey design methodology and abortion stigma. Despite this extended mixed‐methods question development process, none of the six approaches we prioritized for testing resulted in improvements in abortion reporting.

These null results cannot and should not be the end of this effort, even as they highlight the pervasive and intractable impact of abortion stigma. The need to improve the measurement of abortion in U.S. surveys is increasingly vital. With the Supreme Court's *Dobbs* decision allowing states to ban abortions, these draconian laws may shift those seeking abortion to providers outside established networks or increase the prevalence of self‐managed abortions obtained without a clinician. Individual‐level surveys will become more important if administrative and provider records represent a declining share of all abortions. Administrative records also do not replace the need for individual‐level survey data, which permit the study of experiences that are not reflected in simple incidence counts. But the challenges to measuring abortion post‐*Dobbs* have increased, as surveys will now be asking respondents to report on potentially illegal behaviors depending on their state of residence. Additionally, with the changing legal environment and heightened public discourse, the sensitivity and stigma around abortion may increase; there is some evidence of reductions in the quality abortion reporting in surveys in recent years, which may further deteriorate.^10^, ^33^ Increasingly, we will need to be concerned about not only the share of abortions reported in surveys, but biases in differentially complete reporting patterns. New state laws make current survey approaches to measuring abortion even less tenable.

Despite this study's null results, it provides a promising path for future efforts, as a “proof of concept” for testing new approaches in a cheaper, faster, and more flexible format than embedding experimental changes in the NSFG or other national surveys. Working with NORC, we were able to field a large national survey that was rapid, nimble for experimentation, and relatively low‐cost. The ability to field large online surveys with embedded experiments is increasingly available through several different survey firms. Further research can build from this and continue to test alternate approaches and try to move the field forward in how we measure abortion.

Despite its many strengths, this analysis faced several limitations. The addition of a nonprobability sample to the AmeriSpeak® panel provided the advantage of cost‐effectively increasing sample size. However, while we used enrollment targets to ensure a more representative sample in the nonprobability sample, this approach may have unobserved selection biases, impacting the generalizability of results. Still, these analyses indicate no difference in abortion reporting between the probability and nonprobability samples, which suggests the sampling mode was not associated with reporting patterns. In addition, although we had relatively large sample sizes, the study was not powered to detect slight differences between experimental arms; consequently, we could have missed minor true treatment effects of the tested interventions. However, given the scale of underreporting in national surveys, identifying marginal improvements would have limited value for future survey design.

While nonbinary respondents and transgender men were included in this study, we developed the questions from formative research that did not center their experiences; more focused work in this area could be valuable. This analysis also did not engage with the experiences of non‐English speakers or cisgender men. Although there is evidence that abortion is also a stigmatized experience for cisgender men and that they underreport their partners' abortions,^34^ they were not included in much of the formative research or the survey experiment. The experiment was conducted only in English, as there is a need to develop questions in Spanish that are reflective of potentially differentiated reporting issues for Spanish speakers and different understandings and experiences of abortion stigma.^59^ Finally, the models do not include interactions between Factor 1 and Factor 2 (and were not adequately powered to detect interaction effects); as a result, the ATE for any given question wording experiment in Factor 2 (for example) is technically a weighted average of the effect when placed at the beginning of the survey and the effect when placed at the end of the survey.^60^ However, this combined effect is, in fact, the estimate of interest, as it mirrors the real context of question changes in surveys: Any given intervention in question wording or placement is almost always embedded in surveys with multiple other factors that may influence reporting.

We were not able to compare the lifetime estimates of abortion in the 2021 GSRHE to an external “gold standard.” Previous work has constructed synthetic cohorts to estimate that approximately one in four women will have abortions in their lifetime, given current age‐specific rates.^61^ This is likely a substantial underestimate of the proportion of respondents in our sample who have had an abortion in their lifetime, however. Many respondents have spent most of their reproductive years in time periods in which age‐specific abortion rates were substantially higher; however, this is mitigated somewhat by the fact that observations are right censored in our data. We believe that the 16% of respondents reporting an abortion is likely a substantial underreport of the true value of the lifetime prevalence of abortion. Still, it is reassuring that the 2021 GSRHE produced similar estimates of lifetime abortion prevalence and demographic differentials as the NSFG, indicating that the online survey did no worse than the NSFG.

This study focused on two approaches: question placement and question design. The latter included testing an alternative to the question introduction and different wordings and formats of the abortion item itself. The experimental approaches were relatively conservative and built directly from existing design approaches. Evidence that these relatively modest changes did not yield positive results suggests more radical changes may be necessary. While further work on these question approaches could be valuable, it is also worthwhile to investigate other potential influences on reporting, such as incentives paid to the respondents and interviewer effects.^62^, ^63^

This study demonstrates that abortion stigma undermines survey research on abortion and related experiences. However, it is essential that we do not lose sight of how abortion stigma harms people seeking and having abortions.^16^ Stigma can create barriers to obtaining abortion care and increase inequities in health; in a 2019 statement the American College of Obstetrics and Gynecology (ACOG) and 11 other leading reproductive health care organizations affirmed the importance of abortion access and called for eliminating abortion stigma as a barrier to care.^64^ Advocacy and public health efforts on this front include programs such as We Testify (www.wetestify.org) and #ShoutYourAbortion (shoutyourabortion.com), which encourage sharing abortion stories to reduce stigma. Yet, these efforts must push against massive headwinds, as efforts to restrict abortion access in the US gain ground. While overcoming abortion stigma is key to improving survey research on abortion, it is central to supporting people seeking abortions. Abortion data is critical for understanding all pregnancy experiences, not just births. Despite this, the NSFG warns researchers from using its abortion data because of incomplete reporting.^9^ Other surveys are not immune from these data quality issues. Improving abortion data is crucial to efforts to study and improve pregnancy‐related health outcomes and support reproductive health. Researchers need to recognize the flaws and limits of current data collection and seek to develop new and improved abortion measurement.

## FUNDING INFORMATION

Research reported in this publication was supported by the Eunice Kennedy Shriver National Institute of Child Health & Human Development of the National Institutes of Health under Award Number R01HD084473. The content is solely the responsibility of the authors and does not necessarily represent the official views of the National Institutes of Health.

## Appendix

**TABLE A1:** Factor 2 question wording

Condition	Question wording
Control	Sometimes women are reluctant to tell an interviewer about some of their pregnancies, especially those pregnancies that ended in abortion or with babies they no longer live with. In the next set of questions, please account for all of your pregnancies. In your lifetime, how many pregnancies did you have that resulted in live birth, that is, a baby born alive? Number _____ In your lifetime, how many pregnancies did you have that ended in miscarriage, stillbirth, or ectopic pregnancy? Number _____ In your lifetime, how many pregnancies did you have that ended in abortion? Number _____ [If 1+ abortions]: In the past 5 years, how many pregnancies did you have that ended in abortion? Number _____
Order	Sometimes women are reluctant to tell an interviewer about some of their pregnancies, especially those pregnancies that ended in abortion or with babies they no longer live with. In the next set of questions, please account for all of your pregnancies. In your lifetime, how many pregnancies did you have that resulted in live birth, that is, a baby born alive? Number _____ In your lifetime, how many pregnancies did you have that ended in abortion? Number _____ In your lifetime, how many pregnancies did you have that ended in miscarriage, stillbirth, or ectopic pregnancy? Number _____ [If 1+ abortions]: In the past 5 years, how many pregnancies did you have that ended in abortion? Number _____
Level of detail	Sometimes women are reluctant to tell an interviewer about some of their pregnancies, especially those pregnancies that ended in abortion or with babies they no longer live with. In the next set of questions, please account for all of your pregnancies. In your lifetime, have you had a pregnancy that resulted in a live birth, that is, in a baby born alive? (yes/no) In your lifetime, have you had a pregnancy that ended in miscarriage, stillbirth, or ectopic pregnancy? (yes/no) In your lifetime, have you had a pregnancy that ended in abortion? (yes/no) [If yes to abortion]: In the past 5 years, how many pregnancies did you have that ended in abortion? Number _____
Introduction	The following questions will help to improve family planning and health services. In your lifetime, how many pregnancies did you have that resulted in live birth, that is, a baby born alive? Number _____ In your lifetime, how many pregnancies did you have that ended in miscarriage, stillbirth, or ectopic pregnancy? Number _____ In your lifetime, how many pregnancies did you have that ended in abortion? Number _____ [If 1+ abortions]: In the past 5 years, how many pregnancies did you have that ended in abortion? Number _____
Context	In your lifetime, have you received any of the following health services from a doctor or other medical care provider? A pregnancy test (yes/no) An abortion (yes/no) An IUD (yes/no) A Pap test or pelvic exam (yes/no) A breast exam (yes/no) A test for a sexually transmitted disease (STI) (yes/no) [If yes to abortion]: In the past 5 years, how many pregnancies did you have that ended in abortion? Number _____ In your lifetime, how many pregnancies did you have that resulted in live birth, that is, a baby born alive? Number _____ In your lifetime, how many pregnancies did you have that ended in miscarriage, stillbirth, or ectopic pregnancy? Number _____
Graduated sensitivity	Consider if each of the statements below applies to you. Please choose “yes” or “no” for each statement. I have a friend or family member who has had an abortion. I have been pregnant and thought about having an abortion. I have looked online for information about abortion for myself or another person. I have made an appointment for an abortion at a healthcare facility. I have had an abortion. [If yes to abortion]: In the past 5 years, how many pregnancies did you have that ended in abortion? Number _____ In your lifetime, how many pregnancies did you have that resulted in live birth, that is, a baby born alive? Number _____ In your lifetime, how many pregnancies did you have that ended in miscarriage, stillbirth, or ectopic pregnancy? Number _____

Abbreviation: IUD, intrauterine device.

**TABLE A2:** Percentage distribution of respondents according to demographic characteristics for each study arm, 2021 GSRHE

Characteristics		1A2A (*n* = 540) (%)	1A2B (*n* = 541) (%)	1A2C (*n* = 532) (%)	1A2D (*n* = 533) (%)	1A2E (*n* = 531) (%)	1A2F (*n* = 526) (%)	1B2A (*n* = 564) (%)	1B2B (*n* = 540) (%)	1B2C (*n* = 542) (%)	1B2D (*n* = 571) (%)	1B2E (*n* = 557) (%)	1B2F (*n* = 559) (%)
Age	18–19	2	2	2	2	4	3	3	3	2	3	1	3
	20–24	12	13	12	14	10	13	10	9	11	9	11	11
	25–29	18	19	15	18	18	18	14	18	16	17	16	16
	30–34	21	23	20	19	20	18	20	17	21	21	20	22
	35–39	21	18	18	18	19	20	21	21	20	19	24	20
	40–44	14	16	19	15	18	14	18	19	17	16	15	16
	45–49	12	10	14	14	12	13	14	13	14	15	13	12
Race/ethnicity	Non‐Hispanic White	54	55	56	56	56	57	55	57	58	58	57	58
	Non‐Hispanic Black	19	15	18	16	19	17	15	15	14	16	15	18
	Hispanic	17	18	15	17	15	16	15	16	16	14	17	13
	Non‐Hispanic other/multiple races	10	12	10	11	10	10	14	11	11	11	12	11
Marital status	Married	45	43	45	46	42	43	47	45	47	51	46	44
	Cohabitating	17	17	15	17	17	18	14	14	15	12	16	13
	Single	38	40	41	37	40	39	39	41	38	36	38	42
Recruitment mode	Probability	49	50	50	49	47	49	49	49	46	49	50	46
	Nonprobability	51	50	50	51	53	51	51	51	54	51	50	54

*Note*: Sample selection was limited to respondents who were aged 18–49, were assigned female at birth, and had ever had penile‐vaginal sex.

**TABLE A3:** Adjusted odds ratios from logistic regression predicting reporting ever having had an abortion from experimental conditions and demographic controls, 2021 GSRHE

		Adjusted odds ratio	95% CI	
Question placement	Early (ref)	1.00		
	Final	1.00	0.88	1.15
Question wording	Control (ref)	1.00		
	Order	1.05	0.84	1.32
	Level of detail	1.02	0.82	1.28
	Introduction	0.83	0.66	1.05
	Context	0.80	0.63	1.01
	Graduated sensitivity	0.97	0.77	1.22
Age	18–19	1.09	0.62	1.91
	20–24 (ref)	1.00		
	25–29	1.52	1.12	2.07
	30–34	2.23	1.66	2.99
	35–39	2.79	2.08	3.74
	40–44	3.18	2.35	4.28
	45–49	3.69	2.71	5.01
Race/ethnicity	Non‐Hispanic White (ref)	1.00		
	Non‐Hispanic Black	1.75	1.46	2.10
	Hispanic	1.82	1.52	2.19
	Non‐Hispanic other/multiple races	1.07	0.85	1.35
Marital status	Married (ref)	1.00		
	Cohabitating	1.49	1.21	1.82
	Single	1.36	1.16	1.58
Recruitment mode	Probability (ref)	1.00		
	Nonprobability	1.13	0.99	1.30
